# Saturn’s near-equatorial ionospheric conductivities from *in situ* measurements

**DOI:** 10.1038/s41598-020-64787-7

**Published:** 2020-05-13

**Authors:** O. Shebanits, L. Z. Hadid, H. Cao, M. W. Morooka, G. J. Hunt, M. K. Dougherty, J.-E. Wahlund, J. H. Waite, I. Müller-Wodarg

**Affiliations:** 1 Blackett Laboratory, Imperial College London, London, UK; 2 Swedish Institute of Space Physics, Uppsala, Sweden; 3 ESA/ESTEC, Noordwijk, Netherlands; 4 Department of Earth and Planetary Sciences, Harvard University, Cambridge, USA; 5 Division of Geological and Planetary Sciences, California Institute of Technology, Pasadena, USA; 6 Southwest Research Institute, Space Science and Engineering Division, San Antonio, USA

**Keywords:** Space physics, Plasma physics, Planetary science

## Abstract

Cassini’s Grand Finale orbits provided for the first time *in-situ* measurements of Saturn’s topside ionosphere. We present the Pedersen and Hall conductivities of the top near-equatorial dayside ionosphere, derived from the *in-situ* measurements by the Cassini Radio and Wave Plasma Science Langmuir Probe, the Ion and Neutral Mass Spectrometer and the fluxgate magnetometer. The Pedersen and Hall conductivities are constrained to at least 10^−5^–10^−4^ S/m at (or close to) the ionospheric peak, a factor 10–100 higher than estimated previously. We show that this is due to the presence of dusty plasma in the near-equatorial ionosphere. We also show the conductive ionospheric region to be extensive, with thickness of 300–800 km. Furthermore, our results suggest a temporal variation (decrease) of the plasma densities, mean ion masses and consequently the conductivities from orbit 288 to 292.

## Introduction

A planet’s ionosphere is an ionized upper layer of its atmosphere. If the host planet has a magnetosphere, the ionosphere serves as a coupling between the atmosphere and magnetosphere, transferring energy and momentum. Traditionally, an ionosphere is thought to consist only of positive ions and electrons. In Saturn’s case, however, ring particles (dust grains) falling in from the rings absorb the electrons (depleting their densities by more than 80%) and create a layer around the equator with enhanced ion densities^1^. This layer plays an important role in electrodynamics between Saturn’s ionosphere and magnetosphere – the interhemispheric field-aligned currents detected inside the gas giant’s rings^2,3^ must close by means of ionospheric currents. These currents depend on the dimensions of the conductive (dynamo) region and the electrical conductivity of Saturn’s ionosphere.

Prior to the Cassini Grand Finale our knowledge of Saturn’s ionosphere was based on remote sensing by Pioneer 11, Voyagers 1&2 and Cassini. This gave insights into the dawn/dusk regions from radio occultations^4–7^, peak electron densities from radio emissions by Saturn’s lightning^8,9^ and mid-latitude regions by ground-based observations of the atmospheric infrared emissions^10^. Remote sensing provides estimates of the general properties of the ionosphere, including multiple dawn/dusk electron density peaks below 2000 km altitude^5,6^, and is a great complement to the orbit-limited *in-situ* measurements. However, the charged dust grains in Saturn’s equatorial ionosphere^1^ cannot be measured remotely. It should also be noted that the existing remote sensing data do not cover the dayside regions where the *in-situ* measurements were performed (and did not occur at the same epoch), hence a direct comparison is not straightforward.

In this study we quantify the intrinsic ionospheric characteristics that constrain ionospheric currents – the Pedersen and Hall electrical conductivities and the conductive dynamo region. To this end, we utilize the dataset from the Cassini mission, namely the Grand Finale’s last 6 orbits (numbered 288–293), during which the Cassini spacecraft sampled the top ionosphere of Saturn *in-situ* for the first time. The underlying dataset is from the *in-situ* measurements by Radio and Plasma Wave Science Langmuir Probe (RPWS/LP), Ion and Neutral Mass Spectrometer (INMS), and Cassini fluxgate magnetometer (MAG).

**Figure 1:**
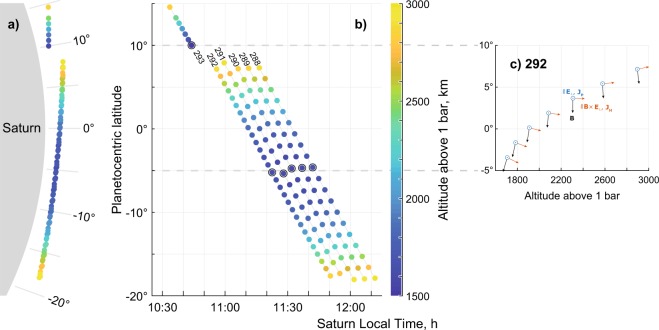
Saturn’s ionosphere spatial coverage map for orbits 288–292 and the Final Plunge (293), as seen from the dawn direction (**a**, to scale with Saturn shown in grey) and in latitude versus local time (**b**). Dots mark the RPWS/LP sweeps, black circles mark the closest approach for respective orbits and the altitude is colour-coded. Panel c: Magnetic field (*B*) direction for this region on example of orbit 292 (${B}_{phi}\ll {B}_{r},{B}_{\theta }$) as well as direction of dayside equatorial Pedersen current (in blue, parallel to electric field ${E}_{\perp }$, eastward or westward) together with the direction of equatorial Hall current (in red, $\parallel B\times {E}_{\perp }$, upward or downward) that completes the orthogonal right-hand system. Note that every second datapoint in panel c has been omitted to reduce clutter.

The orbital geometry limits coverage to the dayside near-equatorial ionosphere: 10:50–12:17 Saturn Local Time (SLT), 10°N–20°S planetocentric latitude (Fig. 1 panels **a** and **b**). The altitude coverage is limited to the closest approach of the Cassini spacecraft, ~1570–1720 km above the 1 bar pressure level. To define the artificial 1 bar “surface” we use the gravity model derived from the Cassini measurements^11,12^.

The ionospheric conductivity (and current) vectors are defined as follows (near-equatorial case shown in Fig. 1c for reference). The Pedersen conductivity is orthogonal to the magnetic field and parallel to the electric field (blue vector in Fig. 1c), the Hall conductivity is orthogonal to both magnetic and electric fields (red vector) and the magnetic field parallel conductivity completes the set (black vector).

The ionospheric conductivities peak in a conductive region of an ionosphere known as a dynamo region. It is defined by the frequencies of momentum transfer collisions ($\nu$) and gyrofrequencies ($\Omega$)^13^: the motion of ions (positive and negative) is disturbed mainly by collisions with neutrals (${\nu }_{i} > {\Omega }_{i}$) while the electrons can still ${\boldsymbol{E}}\times {\boldsymbol{B}}$ drift (i.e., ${\nu }_{e} < {\Omega }_{e}$) and thus form ionospheric currents (in Saturn’s ionosphere, $16.4\le |{\boldsymbol{B}}|\le 18.6$ µT).

The necessary parameters to derive the conductivities are the plasma densities (${n}_{s}$) and masses (${m}_{s}$) the neutral (${H}_{2}$) densities and the magnetic field. The neutral densities are measured by the INMS and the magnetic field by the Cassini magnetometer. The electron densities and temperatures are measured by the RPWS/LP. The positive and negative ion/dust densities can be derived from the RPWS/LP measurements, given mass distributions^14^. However, with the Cassini Plasma Spectrometer (CAPS) shutdown in 2012, a detailed mass distribution of the negative ions or dust grains is not available for the Grand Finale orbits. The RPWS/LP sweep analysis is therefore carried out assuming only positive ions^1^, which in the presence of a significant amount of negative ions (and dust) gives a *lower limit* of the charge density and mean mass of the positive ions^14,15^ (see Methods section). The charge densities of ions in regions with an electron-depleted ion-ion (dusty) plasma are also expected to be enhanced due to the lack of ion-electron recombination^16^.

## Results

### Impact of heavy charge carriers

**Figure 2:**
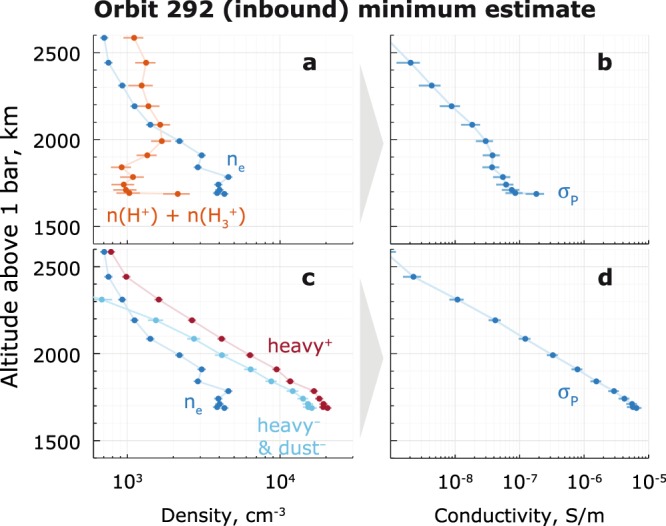
Orbit 292 example: (**a**) the light ion (in red, $n({H}^{+})+n({H}_{3}^{+})$, from INMS, 21% standard error17) and electron (${n}_{e}$) densities (from RPWS/LP, 6.1% standard error1); (**b**) the resulting Pedersen ${\sigma }_{P}$ conductivity. Panels c and d show the same *in-situ* data but with the heavy positive ions (dark red) and the heavy negative ions/dust (cyan) added (from RPWS/LP, 6.1% standard error).

The importance of the heavy positive ions and negative ions/dust grains is illustrated on the example of orbit 292 (Fig. 2). Using only light ions (${H}^{+}$ and ${H}_{3}^{+}$ from INMS^17^) and electrons (from RPWS/LP^1,18,19^) yields Pedersen conductivity (${\sigma }_{P}$) on the order of 10^−7^ S/m (Fig. 2b). As a side note, $n({H}^{+})+n({H}_{3}^{+}) > {n}_{e}$ above 2100 km due to INMS seemingly overestimating the light ion densities at these altitudes^17^, but forcing the quasi-neutrality is not necessary for this example. Now, using RPWS/LP^1^ profiles, which also include the heavy positive ions and dust grains, yields a *minimum* estimate of the Pedersen conductivity that is *two* orders of magnitude higher (Fig. 2d). This is because below ~2100 km altitude, the heavy ionospheric species outnumber the electrons and light ions by factors up to 4 and 18, respectively (Fig. 2c), translating into electron depletion of >60% (${n}_{e}/{n}_{i}\le 0.4$)^1^. The heavy positive ion profiles here are only from the RPWS/LP-derived densities. A similar increase of Pedersen conductivity was also shown for the dusty plasma of the Enceladus plume^20,21^.

### Conductivities of Saturn’s near-equatorial ionosphere

**Figure 3:**
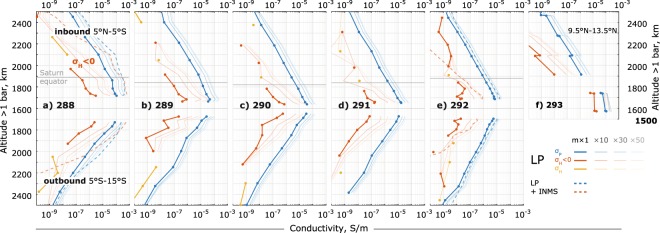
Minimum estimates of the Pedersen ${\sigma }_{P}$ (blue) and Hall ${\sigma }_{H}$ (red <0 and yellow >0) conductivities for orbits 288–292 (**a–e**) and the Final Plunge (**f**). The plots are in altitude, along inbound (top) and outbound (bottom) trajectories, gray horizontal lines show crossings of Saturn’s equatorial plane. See also legend in panel a. The outbound trajectories have reversed altitude axis for easier comparison with inbound (and roughly representing the orbits as shown in Fig. 1a). Solid lines are derived from RPWS/LP ion data alone. Dashed lines (**a** 288 and **e** 292 only) also have INMS ${H}^{+}$ and ${H}_{3}^{+}$ densities, excluding the regions with densities exceeding electron densities^17^. The fainter lines show the profiles for mass factors 10, 30 and 50, for reference. Errors on the Pedersen conductivity are smaller than the markers. Errors on the Hall conductivities are 2 orders of magnitude larger than the values and are omitted here to avoid clutter (see the Methods section for details).

Figure 3 shows the *minimum* estimates of Pedersen and Hall conductivities. The outbound plots have inverse y-axis so that whole trajectories roughly represent latitudinal profiles. The faded lines show some profiles for higher mass factors to illustrate the upper constraint due to particle mass. The crossing of Saturn’s equatorial plane is indicated by horizontal gray lines. Orbits 288 and 292 also show profiles derived with INMS ${H}^{+}$ and ${H}_{3}^{+}$ densities included (dashed lines). That is, the plasma composition is ${H}^{+}$, ${H}_{3}^{+}$, heavy positive species and heavy negative species – the total densities are still the RPWS/LP densities. These profiles highlight the anomaly of orbit 288 – for orbit 292 including the lighter ions produces an overall increase of factor ~2. The increase is due to the momentum transfer collision frequencies being weighted towards the lighter species as collisions with much heavier particles are simply inefficient in transferring momentum (see Methods section). The largest impact of including the light ions is seen in the Hall conductivities, suggesting for orbit 288 that the Hall conductivities may be larger than Pedersen conductivities already at 1900 km above 1 bar, which in turn is indicative of a current layer. However, the azimuthal magnetic field measurements indicate that ionospheric currents are below the spacecraft^3^.

The fainter profiles in Fig. 3 show the dependence of the conductivities on heavy ion and dust grain masses. From bright to faint, the RPWS/LP-derived mean positive ion masses are scaled as $1\times {m}_{i}$, $10\times {m}_{i}$, $30\times {m}_{i}$, and $50\times {m}_{i}$ (see legend), where $1\times {m}_{i}$ represents the minimum estimate. Based on the Titan’s case of dusty ionosphere^14^, the true mean masses of ions and dust grains may be factor ~2 larger than the RPWS/LP-derived masses. Such a parameter study shows that even the influence of the lighter ions (dashed lines) may be overshadowed by the heavy charge carriers.

The orbits 288–293 included in this study only cover the near-equatorial dayside ionosphere, but we note that similar conditions may also exist at higher latitudes (up to ±50°), due to the rings’ dust particles falling in along the field lines^22^.

For all orbits, the Pedersen conductivities at closest approach (~1570–1720 km above 1 bar) are between ${10}^{-4}$ and $5\times {10}^{-4}$, which is at least two orders of magnitude larger than expected based on the radio occultations at these altitudes^23^, even for our most conservative estimate. This fact cannot be overlooked even though the available radio occultations cover different regions of Saturn’s ionosphere.

### Temporal trends

**Figure 4:**
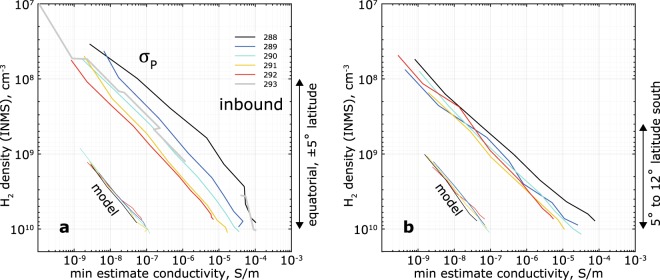
Minimum estimates of inbound (**a**) and outbound (**b**) Pedersen conductivities plotted in the measured neutral atmosphere density (${{\boldsymbol{H}}}_{2}$), illustrating the decrease of the conductivities. The latitude range for the decrease in both parts of the orbits is for Rev 288–292 only, as Rev 293 terminated at 10° north (Fig. 1a). The profiles marked “model” are the Pedersen conductivities based on the GCM^24^ and are shown for reference.

**Figure 5:**
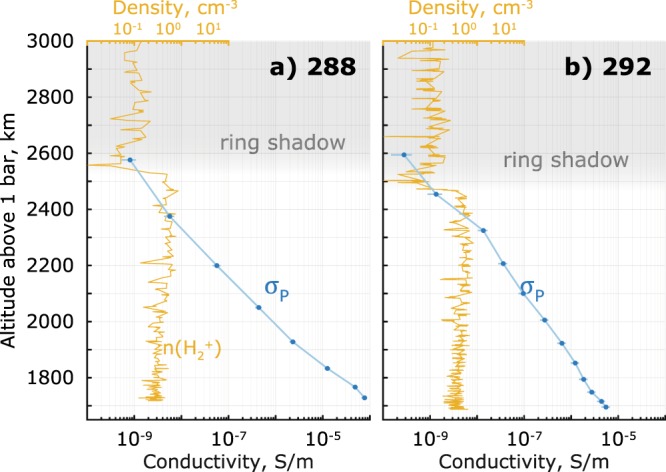
Pedersen conductivity (bottom axes) for outbound parts of orbit 288 (**a**) and 292 (**b**), plotted versus altitude together with the INMS measured ${H}_{2}^{+}$ densities (top axes) as the ring shadowing indicator – the ring shadowing is seen as the sharp drop in the ${H}_{2}^{+}$ densities around 2500 km, marked by shaded areas.

The Pedersen conductivities consistently decrease from orbit 288 through 292. This decrease does not depend on the variabilities in the background neutral atmosphere, as evident from Fig. 4. Furthermore, the Pedersen conductivities along the Cassini trajectory derived from the GCM^24^ (marked “model”) do not exhibit a similar variability. This excludes causes like changes in photoionization or the local time shift along and between the orbits; another evidence is that the profiles of the very short-lived ${H}_{2}^{+}$ ions barely change between orbits 288 and 292 (Fig. 5). Interestingly, conductivity profiles from orbits 291, 292 and 293 are very similar even though 293 terminated at much higher latitudes (≈10°N), suggesting that the lower profiles correspond to baseline levels and the higher ones are the anomalies.

These trends propagate of course from the corresponding decline in the heavy ion densities. The cause is yet unclear. While many instruments operated outside of their design parameters during the Grand Finale orbits and instrumental artifacts are a concern for RPWS/LP^1^ and INMS^17^ derived ion densities (as mentioned above), it should be noted that the plasma density measurements by three different instruments are in a good agreement^17,19^. Moreover, instrumental artifacts seen by the RPWS/LP for orbit 288 were clearly identified and removed from analysis (and did not present for the subsequent orbits)^1^.

One plausible explanation for the decreasing trend in the derived conductivities is that we are seeing a temporal change (decrease) of the dust influx affecting the plasma densities, over the course of about 1 month (the time between orbits is nearly constant, ≈6.5 days). However, the neutral dust influx estimated from the INMS measurements for the orbits 290 through 292 shows a maximum influx for orbit 291 – i.e., no subsequent decrease is evident, although a factor 2 fluctuation is notable^17^. Indeed, the measured high variability^18,19^ of the Kronian ionosphere leaves little to no reasons to assume a steady state in this context, but proving or disproving this hypothesis requires further investigation.

Another explanation may be atmospheric waves shifting the whole atmosphere and ionosphere in altitude by ±10% of the background ${H}_{2}$ densities^24^, or 30–50 km with respect to the 1 bar level. However, in such a scenario the decreasing trend should not be visible in a plot versus measured ${H}_{2}$ densities (Fig. 4).

### Spatial trends

Apart from the overall decrease in conductivities, there is a latitudinal (inbound-outbound) asymmetry in the conductivity profiles (Fig. 3, Fig. 4), also propagating from the measured ion densities. This may in part be attributed to the inbound trajectories being much closer to the subsolar point (12 SLT, ≈ 27° N latitude) than the outbound ones (Fig. 1a), although again, the model profiles in Fig. 4 (which include photoionization) exhibit much smaller variability.

One of the possible causes is a latitude-dependent dust influx: the inbound conductivity profiles cover equatorial region (±5° latitude) which has a much larger (neutral) dust influx^17^, while the outbound profiles cover southern near-equatorial region (>5°S, down to the ring shadow at ≈20°S), with a relatively smaller (charged) dust influx at >15°S^22^. This influx of charged dust will increase the conductivities (Eq. (1)) on the edge of the covered region – around 2500 km altitude on the outbound trajectories.

Another possibility is the ring shadowing of the outbound profiles. This has been investigated by comparing the RPWS/LP plasma density measurements and densities of the short-lived ${H}_{2}^{+}$ ions (produced by photoionization) measured by the INMS^25^. For the orbit 288 the shadow begins at 2500 km altitude (latitude 15° south) as indicated by a sharp drop in the ${H}_{2}^{+}$ densities (Fig. 5a), and slightly lower for the orbit 292 (Fig. 5b). This again does not match the asymmetry of the conductivity profiles.

The most plausible explanation is therefore that the ion densities in the equatorial region are enhanced in the presence of neutral dust (which gets ionized locally by electron attachment), similar to Titan’s ionosphere^26–28^.

### Dusty plasma peak

In the dusty plasma of Titan’s ionosphere, the ion densities have a second, larger peak at lower altitudes^28^. The ion densities are enhanced in electron-depleted regions of ionosphere due to ion-ion recombination being much slower than electron-ion one^26^, as mentioned above. If Saturn’s ionosphere is similar in this regard, Pedersen and Hall conductivities should also have a second, larger peak at altitudes below ~1500 km since both conductivities scale linearly with the ion densities. We want to stress that while the available Pedersen conductivities from radio occultations^23^ do have a larger peak at about 1000 km altitude, they are based on the electron densities and their peaks are fundamentally different from peaks due to dusty plasma.

### Reverse Hall effect

Note that the Hall conductivities in Fig. 3 are mostly negative. This is expected for a dusty plasma^20,21^. The negative Hall conductivity of the dusty plasma means that the Hall current is reversed. A simplified explanation for this is as follows. Traditionally, a Hall current is associated with electrons (negative), the lighter and more mobile component of a plasma, while the much heavier ions (positive) contribute very little. In a dusty plasma, however, the electrons are depleted^1,14,19,29–31^ and the dominant lightest species are instead positive ions, while the heavier charge carriers are negative. Such a role reversal is mirrored in the direction of the current. This reversal of the Hall conductivities (and extended dynamo region) also adds complexity to the detection of ionospheric currents at Saturn. In particular, the equatorial electrojet is associated with Cowling conductivity, which is defined as ${\sigma }_{P}+{\sigma }_{H}^{2}/{\sigma }_{P}$. In fact, measurements in Earth’s equatorial ionosphere^32^ have shown that dust grains introduced by meteor ablation deplete the electron densities and decrease or indeed reverse the Hall and Cowling conductivities, a striking similarity to the effect of equatorial ring rain into Saturn’s ionosphere.

### Conductances

The height-integrated conductivities for the respective profiles are given in Table 1 with the associated combined measurement errors. We calculate the conductances for three mass factors, for $1\times {m}_{i}$ (minimum), $10\times {m}_{i}$ and $20\times {m}_{i}$ to again show the dependency on the charge carrier mass. Note that the integrations (like measurements) are cut at the closest approach and are therefore *smaller* than the total ionospheric conductances. In particular, for the orbit 293 that is closer to the sub-solar point, the Pedersen conductance above 1570 km is 15.9 S, a factor two larger compared to the conductance from the full radio occultation profile (~1000 km *deeper*)^23^. We therefore conclude that for the coupling of Saturn’s magnetosphere and ionosphere (i.e., closure of currents, energy transport) the dusty plasma must be considered if the equatorial ionosphere is involved. For instance, in the context of ionospheric heating by the Joule effect, for a set electric field (${\boldsymbol{E}}$) a higher equatorial conductivity translates into proportionally larger current density (${\boldsymbol{J}}$) and heating, since the generated power is ${\boldsymbol{J}}\cdot {\boldsymbol{E}}={E}^{2}\sigma$. However, concluding on the impact of the dust grains on the ionospheric currents at Saturn requires extensive modelling as the presented *in-situ* measurements do not provide sufficient vertical coverage.

**Table 1:** Height-integrated conductivities (in S) for the mass factors 1, 10 and 20 (applied to the mass from RPWS/LP). Note that these represent the *in-situ* covered altitudes only.

		Pedersen		Hall	
		inbound	outbound	inbound	outbound
**288**	1 × m_i_	**13.9 ± 1.7**	5.4 ± 0.7	**−0.1 ± 20.6**	−0.1 ± 6.6
	10 × m_i_	**39.2 ± 4.5**	15.3 ± 1.9	**−1.4 ± 20.7**	−1.6 ± 6.6
	20 × m_i_	**54.3 ± 6.0**	21.0 ± 2.4	**−2.9 ± 20.8**	−3.5 ± 6.7
**289**	1 × m_i_	**5.8 ± 0.8**	1.6 ± 0.3	**−0.1 ± 12.3**	0.0 ± 5.4
	10 × m_i_	**14.9 ± 1.8**	3.4 ± 0.5	**−0.6 ± 12.3**	−0.1 ± 5.4
	20 × m_i_	**20.5 ± 2.4**	4.5 ± 0.6	**−1.3 ± 12.3**	−0.1 ± 5.4
**290**	$1\times {{\rm{m}}}_{{\rm{i}}}$	**3.4 ± 0.5**	2.5 ± 0.4	**0.2 ± 9.1**	0.0 ± 5.2
	$10\times {{\rm{m}}}_{{\rm{i}}}$	**7.9 ± 1.0**	5.4 ± 0.8	**−0.1 ± 9.1**	−0.2 ± 5.2
	$20\times {{\rm{m}}}_{{\rm{i}}}$	**10.7 ± 1.4**	7.2 ± 1.0	**−0.4 ± 9.1**	−0.5 ± 5.2
**291**	$1\times {{\rm{m}}}_{{\rm{i}}}$	**1.5 ± 0.3**	1.1 ± 0.2	**0.0 ± 4.9**	0.0 ± 3.4
	$10\times {{\rm{m}}}_{{\rm{i}}}$	**3.0 ± 0.5**	2.0 ± 0.3	**−0.1 ± 4.9**	0.0 ± 3.4
	$20\times {{\rm{m}}}_{{\rm{i}}}$	**4.0 ± 0.6**	2.7 ± 0.4	**−0.2 ± 4.9**	−0.1 ± 3.4
**292**	$1\times {{\rm{m}}}_{{\rm{i}}}$	**0.8 ± 0.1**	0.5 ± 0.1	**0.0 ± 4.0**	0.1 ± 3.5
	$10\times {{\rm{m}}}_{{\rm{i}}}$	**1.5 ± 0.2**	1.0 ± 0.2	**0.0 ± 4.0**	0.1 ± 3.5
	$20\times {{\rm{m}}}_{{\rm{i}}}$	**1.9 ± 0.3**	1.2 ± 0.2	**0.0 ± 4.0**	0.1 ± 3.5
**293**	$1\times {{\rm{m}}}_{{\rm{i}}}$	**15.9 ± 0.4**		**−2.3 ± 5.1**	
	$10\times {{\rm{m}}}_{{\rm{i}}}$	**36.3 ± 0.4**		**−17.3 ± 5.1**	
	$20\times {{\rm{m}}}_{{\rm{i}}}$	**41.8 ± 0.4**		**−26.3 ± 5.1**	

### Dynamo Region

**Figure 6:**
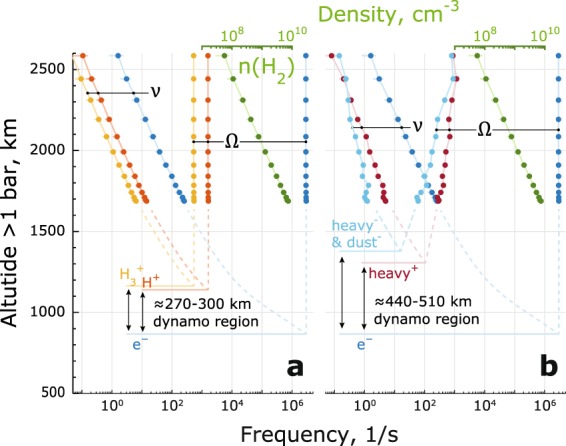
Orbit 292 (inbound) example of the dynamo region boundaries derived with only the light ion species (**a**) and with heavy charge carriers (**b**). Minimum mass derived from the RPWS/LP is used here (see Methods: Masses of positive ions and negative dust grains). The boundaries are defined by the momentum transfer collision frequencies $\nu$ and the gyrofrequencies $\Omega$ as ${\nu }_{i} > {\Omega }_{i}$, ${\nu }_{e} < {\Omega }_{e}$. Profiles for ${H}_{3}^{+}$ are shown in yellow, for ${H}^{+}$ in red, for electrons in blue, for heavy negative charge carriers in cyan and for heavy positive charge carriers in dark red. Note that the ${H}_{2}$ densities (green) have x-axis on top. The boundaries are estimated by extrapolating the ${H}_{2}$ densities using a hydrostatic fit (dashed lines).

**Figure 7:**
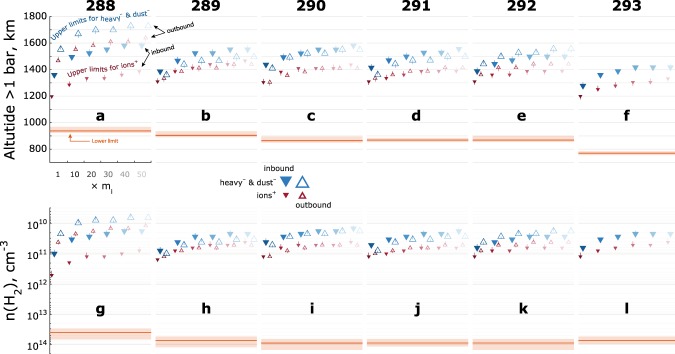
Estimates of the ionospheric conductive (dynamo) region limits for orbits 288–293, plotted in altitude above 1 bar (**a–f**) and ${H}_{2}$ number density (**g–l**). Inbound markers are filled, outbound markers are empty. The larger blue markers show the negative ions and dust grains, the smaller red markers show the positive ions. Brightest colours show the minimum estimate (for the masses derived from RPWS/LP, see the Methods section). Colour gradient shows mass dependency, which is largely due to a lower gyrofrequency of the heavier particles. Red shade shows combined standard error for the lower limit.

The dynamo region (conductive layer of an ionosphere) is defined as ${\nu }_{i,d} > {\Omega }_{i,d}$ (upper boundary) and ${\nu }_{e} < {\Omega }_{e}$ (lower boundary) and therefore is also significantly affected by the presence of the heavy charge carriers. This effect is illustrated in Fig. 6 for orbit 292 (minimum estimate) and in Fig. 7 for all orbits. Since the measurements are limited by the closest approach, the neutral ${H}_{2}$ densities are extrapolated by a hydrostatic fit to the INMS measurements (see Methods for details). The largest associated error for this extrapolation is from the INMS ${H}_{2}$ profiles themselves (≈30% standard error).

Using only the light positive ions results in the dynamo region thickness of ≈270–300 km (Fig. 6a), centered around 1000 km altitude Such a conductive layer is consistent with the altitude typically used for the location of ionospheric currents at Saturn^2,3,33^. Adding the heavy species increases the dynamo region thickness to ≈440–510 km (Fig. 6b). This example also demonstrates that the upper boundary of the dynamo region is not trivial in a multi-species ionosphere, as it is different for each mass group. In absence of mass distributions, we use the dominating heavy positive ions and negative ions/dust grains to define the upper boundary.

A sidenote regarding the small-scale structure in the collision and gyrofrequencies of the heavy species in Fig. 6b, there are two factors at work. Firstly, the RPWS/LP derived positive ion mass does show some structure, which influences the collision frequencies and gyrofrequencies. One should keep in mind, however, that Cassini traverses altitude and latitude simultaneously and the presented profiles are not strictly vertical. Secondly, apart from collisions with neutrals, collisions of positive ions and negative dust grains are also included as they drift in the opposite directions (see also Methods section). Both of these effects are quite small and therefore only add the small-scale variability in the collision and gyrofrequencies of the heavy species.

The estimates of the dynamo region boundaries for all included orbits are shown in Fig. 7, plotted versus altitude (**a**–**f**) and neutral atmosphere (**g**–**l**). The lower limits are marked by red lines (red shade is the combined standard error). The upper limit are marked by triangles (downward and filled for inbound, upward and empty for outbound), red for heavy positive ions, blue for heavy negative ions and dust grains. The upper limit estimates for different mass factors are shown with the fading colour gradient to illustrate the mass dependence: the minimum estimate for $1\times {m}_{i}$ is the brightest and the estimates for $\,10\times {m}_{i}$ through $50\times {m}_{i}$ are successively fainter.

Even for the minimum estimate ($1\times {m}_{i}$), the dynamo region thickness is ~400–500 km. Such an extensive dynamo region provides more ionospheric volume for carrying currents, suggesting that the magnetospheric currents closing through the ionosphere may result in tenuous current densities. This presents a challenge for detecting such currents.

## Conclusions

1. We have shown that even conservative low estimates of Saturn’s ionospheric conductivities (near-equatorial dayside) are at least 10–100 times larger than estimates based on electron densities alone. This increase is due to the presence of dusty plasma. It adds a new level of complexity for the ionosphere-magnetosphere coupling by current systems and must be included in ionospheric models concerning equatorial region.
2. The conductivities decrease from orbit 288 to 290 due to an underlying decrease in the ion densities and masses, suggesting a possible temporal change in the influx of ring dust.
3. The conductivity profiles of orbits 288 through 292 exhibit an inbound-outbound asymmetry (diminishing from orbit to orbit), most likely due to a much larger dust influx around equator (i.e., ring plane).
4. The Hall conductivity is reversed by the presence of the charged dust grains and electron depletion.
5. The ionospheric dynamo region is extended by the dominance of heavy positive ions and negative ions/dust grains, spanning from ≈900 to 1600–1700 km above 1 bar level. This implies a low ionospheric current density near equator.
6. Finally, we would like to again stress that our results represent minimum estimates due to the dependencies on the mean ion masses and ion densities, both of which are likely underestimated by the available analysis of the RPWS/LP Grand Finale data.

## Methods

### Conductivities

The Pedersen (${\sigma }_{P}$), Hall (${\sigma }_{H}$) and magnetic field parallel (${\sigma }_{\parallel }$) conductivities are defined using the conductivity tensor representation in terms of the gyro- and collision frequencies^34^ (amended with the dust component, SI units):

1 $$\begin{array}{rcl}{{\rm{\sigma }}}_{P} & = & {{\rm{\sigma }}}_{e}\frac{{\nu }_{e}^{2}}{{{\rm{\nu }}}_{e}^{2}+{\Omega }_{e}^{2}}+\sum _{d}\,{\sigma }_{d}\frac{{\nu }_{d}^{2}}{{\nu }_{d}^{2}+{\varOmega }_{d}^{2}}+\sum _{i}\,{{\rm{\sigma }}}_{i}\frac{{\nu }_{i}^{2}}{{{\rm{\nu }}}_{i}^{2}+{\Omega }_{i}^{2}}\\ {{\rm{\sigma }}}_{H} & = & \left({{\rm{\sigma }}}_{e}\frac{{{\rm{\nu }}}_{e}{\Omega }_{e}}{{{\rm{\nu }}}_{e}^{2}+{\Omega }_{e}^{2}}+\sum _{d}\,{\sigma }_{d}\frac{{\nu }_{d}{\varOmega }_{d}}{{\nu }_{d}^{2}+{\varOmega }_{d}^{2}}\right)-\sum _{i}\,{{\rm{\sigma }}}_{i}\frac{{{\rm{\nu }}}_{i}{\Omega }_{i}}{{{\rm{\nu }}}_{i}^{2}+{\Omega }_{i}^{2}}\\ {{\rm{\sigma }}}_{\parallel } & = & \frac{{n}_{e}{q}_{e}^{2}}{{m}_{e}{\nu }_{e,tot}}\end{array}$$

The subscripts denote electrons (*e*) positive ions (*i*) and negative ions/dust grains (*d*). The variables are: number density ($n$), charge ($q$), mass ($m$), gyrofrequency ($\Omega$), momentum transfer collision frequencies (${\nu }_{s}$) of species *s* with ${H}_{2}$^34,35^ (${\nu }_{e,tot}$ is for electrons with *all* other species), and ${{\rm{\sigma }}}_{s}=\,{n}_{s}{q}_{s}^{2}{({m}_{s}{{\rm{\nu }}}_{s})}^{-1}$. The dust grains are treated as singly-charged negative ions, with charge densities given by the quasi-neutrality condition, $|{q}_{d}|{n}_{d}=|{q}_{i}|{n}_{i}-|{q}_{e}|{n}_{e}$. To quantify the sensitivity to the charge number of the dust grains, using ${q}_{d}=2{q}_{e}$ will increase the conductivities by a factor of ~3. However, implementing multiply charged dust particles requires knowledge about the mass and charge distribution which is unavailable at the time of writing this article, and we again stress that the derived conductivities represent the *lower limit*.

The inclusion of the dust term in Eq. (1) is required as the near-equatorial ionosphere of Saturn has been shown to be dusty^1^, hosting heavy molecular ions^17,36^ and dust particles that fall in from the rings (with larger particles ablating into smaller)^22,37,38^. In derivation of the conductivities from the momentum equation, only the momentum transfer collisions and the Lorentz force are significant^34^. For the heavy dust grains, the gravity term will be comparable to or larger than the Lorentz term, but will only add drift terms to the currents, not the conductivities.

### Momentum transfer collision frequencies

**Figure 8:**
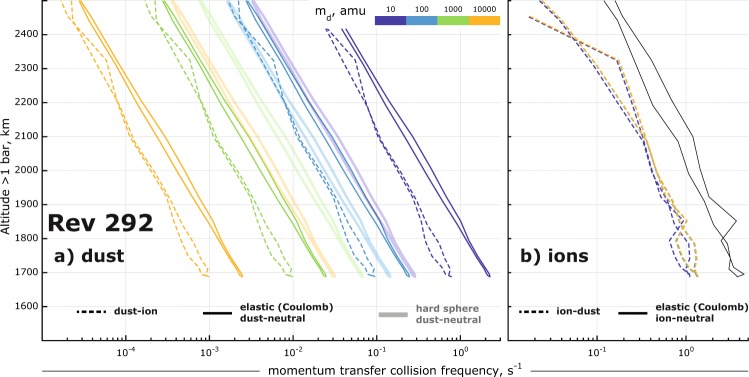
Momentum transfer collision frequencies for dust grains (**a**) and ions (**b**) as a function of dust mass (colour-coded), plotted in altitude for Rev 292. The frequencies of elastic collisions are plotted as thick solid lines, dust-ion and ion-dust collisions (“ion drag”) as dashed lines and the hard-sphere dust-neutral collisions as solid lines.

A comparison of different momentum transfer collision frequencies for the negative ions/dust grains are shown in Fig. 8. The dominant collisions for masses $\le$ 100 amu are elastic (Coulomb) collisions with neutrals (solid lines), however for higher masses the hard-sphere collisions (fainter thick lines) become important.

For ion-neutral collisions, we simplify the expression by Schunk & Nagy^34^ (their equation 4.88) following the Eqs. 8–9 in Banks’ original work^39^:

2 $$\,{\nu }_{in}=2.5879\times {10}^{-9}\frac{{n}_{{H}_{2}}}{{m}_{i}}\sqrt{{Y}_{n}\mu }.$$

Here ${n}_{{H}_{2}}$ is the ${H}_{2}$ number density (cm^−3^), ${Y}_{n}=0.82$ Å^3^ is the ${H}_{2}$ polarizability (Schunk & Nagy’s Table 4.1) and $\mu =\frac{{m}_{1}{m}_{2}}{{m}_{1}+{m}_{2}}$ (in amu) is the reduced mass of the colliding particles. This expression is valid for elastic collisions of ions and neutrals and as such adapted also for the collisions of the negatively charged dust grains and neutrals. Note that Banks writes his equations for the centre of mass, while Schunk & Nagy give them in the lab (ionosphere) frame of reference, the difference being the ${m}_{n}/({m}_{i}+{m}_{n})$ term.

The positive ions and negative dust grains have opposite charge and drift in the opposite directions, creating so-called ion drag^20^. The resulting collisions are elastic (dashed lines in Fig. 8) and for them we use Schunk & Nagy’s expression (their equation 4.142):

3 $${\nu }_{id}=1.27\,{Z}_{d}^{2}{Z}_{i}^{2}\sqrt{\mu }\frac{{n}_{d}}{{m}_{i}{T}_{id}^{1.5}},$$

where Z is charge number, m is mass (in amu), n is density in cm^−3^, and reduced temperature in K is ${T}_{id}=({m}_{i}{T}_{d}+{m}_{d}{T}_{i}){({m}_{i}+{m}_{d})}^{-1}=1$ because ${T}_{i}={T}_{d}$ is assumed. Such an assumption is reasonable in a collision-dominated ionosphere, producing negligible errors because ${\nu }_{di}\ll {\nu }_{dn}$ and ${\nu }_{id}\ll {\nu }_{in}$ (Fig. 8a,b, respectively).

For electron-neutral collisions we use an ${H}_{2}$-specific hard-sphere collision approximation from Schunk & Nagy (their equation 4.156):

4 $${\nu }_{e{H}_{2}}=\frac{8}{3\sqrt{\pi }}\frac{{n}_{{H}_{2}}\,{m}_{{H}_{2}}}{{m}_{e}+{m}_{{H}_{2}}}{\left(\frac{2{k}_{b}{T}_{e{H}_{2}}}{{\mu }_{e{H}_{2}}}\right)}^{0.5}\pi {\sigma }^{2},$$

where ${T}_{eH2}$ is the reduced temperature and ${k}_{b}$ is the Boltzmann constant. The cross-section $\pi {\sigma }^{2}$ is replaced by the recommended^40^ electron-${H}_{2}$ momentum transfer collision cross-section $\sigma ({T}_{e})$. Exploiting the fact that ${m}_{e}\ll {m}_{{H}_{2}}$ we can approximate ${T}_{e{H}_{2}}\approx {T}_{e}$, ${\mu }_{e{H}_{2}}\approx {m}_{e}$ and ${m}_{{H}_{2}}/({m}_{e}+{m}_{{H}_{2}})\approx 1$. Plugging in the constants, Eq. (4) simplifies to:

5 $${\nu }_{e{H}_{2}}\approx 8.2833\times {10}^{13}\,{n}_{{H}_{2}}\sqrt{{T}_{e}}\,\sigma ({T}_{e}),$$

where ${n}_{{H}_{2}}$ is in cm^−3^, temperature is in K and $\sigma ({T}_{e})$ is in cm^−2^. This expression represents Coulomb collisions and as such is also used for the collisions of electrons and negatively charged dust grains.

For the parallel conductivity, the total electron collision frequency is

6 $${\nu }_{e,tot}={\nu }_{ee}+{\nu }_{ei}+{\nu }_{e{H}_{2}},$$

where ${\nu }_{ee}$ is the electron-electron collision frequency and ${\nu }_{ei}$ is the electron-ion collision frequency (Schunk & Nagy’s equations 4.144 and 4.145). In Saturn’s ionosphere ${\nu }_{e{H}_{2}}\gg {\nu }_{ee},{\nu }_{ei}$ for altitudes <3000 km above 1 bar.

Finally, the hard-sphere collision frequency ${\nu {\prime} }_{dn}$ is calculated using Schunk & Nagy’s equation 4.156. This expression has also been used for Enceladus dust grains^20^. For the derivation of conductivities we use the total collision ${\nu }_{i}={\nu }_{in}+{\nu }_{id}$ and ${\nu }_{d}={\nu }_{dn}+{\nu {\prime} }_{dn}+{\nu }_{di}$.

### Dataset and uncertainties

**Figure 9:**
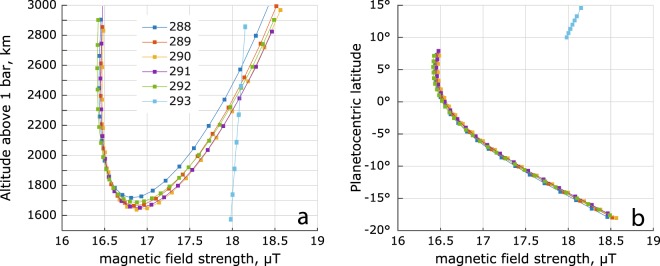
Magnetic field strength for orbits 288 through 293. The 1 s magnetic field data from the MAG instrument has been interpolated to match the times of the RPWS/LP sweeps.

The dataset is composed as follows. The INMS measurements^41–43^ provide profiles (published^17^) of the dominant neutrals (${H}_{2}$) and for orbits 288 and 292, lighter (amu < 4) positive ions ${H}^{+}$ and ${H}^{3+}$. The RPWS/LP measurements^14,30,44,45^ provide the electron temperature and density as well as the total charge density of ions including heavier (amu > 4) components (published^1^). The MAG^46,47^ provides the total magnetic field strength (Fig. 9) for the gyrofrequencies. The spatial resolution for this study is limited by the s/c velocity of 33–34.4 km/s and the temporal resolution of 32–48 s for the RPWS/LP sweeps, see Fig. 1a.

Due to the highly non-linear relationship between the input parameters and the resulting collision frequencies and conductivities, the uncertainties in the results are derived by using Monte-Carlo methods (${10}^{6}$ iterations). All of the measured input parameters are randomly generated from normal distributions based on their respective measurement uncertainties, propagating the errors to the results at each measurement point.

The largest error contribution is from the INMS ${H}_{2}$ profiles with a standard error ≈30% of the density values. The most notable effect the larger errors in the Hall conductivities (${\sigma }_{H} \sim \nu \Omega$) compared to those in the Pedersen conductivities (${\sigma }_{P} \sim {\nu }^{2}$) because $\nu  \sim {n}_{{H}_{2}}$ and ${\nu }_{i,d}\gg {\Omega }_{i,d}$. The uncertainty in ${n}_{{H}_{2}}$ is also the largest contribution to the error in the extrapolation of the INMS ${H}_{2}$ profiles to lower altitudes by means of the hydrostatic fit. For the RPWS/LP ion densities, since the ion current may in some cases have contributions from non-ion sources we use a conservative error estimate of 10% of the density values^1^ (90% confidence), corresponding to the standard deviation of ≈6.1%. The RPWS/LP electron densities derived from the 20 Hz mode have the same error estimation since the methodology is similar. The RPWS/LP electron temperatures have an associated measurement error of 20%^1^ (80% confidence), corresponding to the standard deviation of ≈15.6%. Nevertheless, it should be pointed out that the RPWS/LP plasma densities, the INMS ion densities and the electron density estimates from the upper hybrid emissions in regions without heavy ions (>2500 km altitude) are consistent^17,19,25^. Lastly, the magnetic field strength has the least impact with the standard deviation of ≈0.0087%.

#### Masses of positive ions and negative dust grains

**Figure 10:**
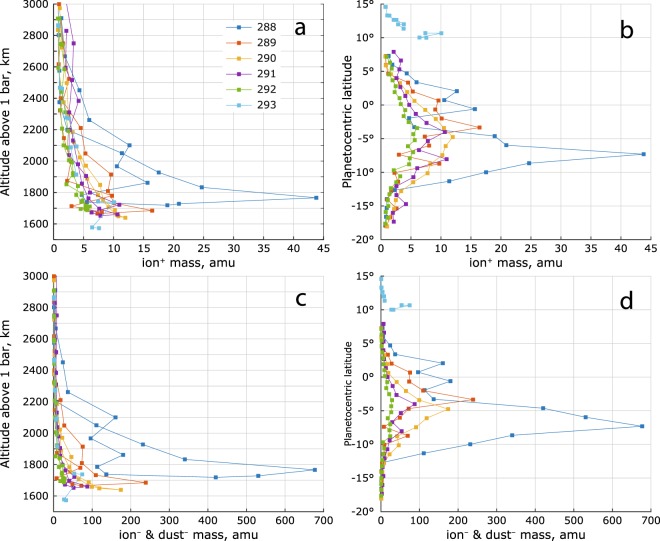
RPWS/LP derived lower estimate of the mean positive ion mass ${m}_{i}$ (panels a and b) and the corresponding lower estimate of the mean negative ion (and dust) mass ${m}_{d}={m}_{i}\,{n}_{i}/{n}_{e}$ (panels c and d). The electron depletion parameter ${n}_{e}/{n}_{i}$ is given in Morooka *et al*., 2019 (their Fig. 6c).

The Cassini measurements show presence of chemically produced heavy molecules^17^ and that the positively charged dust grain counts are proportional to the heavy positive ion densities^37^. This suggests that the mean mass of the dust grains in general should increase towards lower altitudes, similar to Titan’s ionosphere^48^. Furthermore, positive ion mass derived from the RPWS/LP measurements reaches 5–10 amu at closest approach^1^ (Fig. 10), and as mentioned above, this is a lower limit^1,14^. Therefore, we use an empirical *lower constraint* for the mean negative ion/dust mass as ${m}_{d}={m}_{i}{({n}_{e}/{n}_{i})}^{-1}$. This results in ${m}_{d}/{m}_{i}\le 30$ (median ≈ 5 below 2000 km altitude) and is also consistent with an increase of the mean mass of neutrals towards lower altitudes^36^. Such approximation was successfully used to derive *low estimates* of the positive and negative ion densities in similarly dusty ionosphere of Titan^14,27,49,50^.

Our results therefore represent a *lower limit* for the conductivities constrained by the *in-situ* measurements. We note that the conductivities depend linearly on the ion and dust charge densities, allowing a trivial correction following any future investigations of the RPWS/LP measurements during the orbits 288–293. The dependency of the conductivities on the ion and dust mass is roughly linear, propagating from $\nu$, $\Omega$ and ${{\rm{\sigma }}}_{i,d}$ in Eq. (1).

### Dynamo region boundaries

Since the Cassini spacecraft did not traverse the full extent of Saturn’s near-equatorial ionosphere, the boundaries of the dynamo region are estimated by extrapolating the electron-neutral collision frequency towards lower altitudes using the ${H}_{2}$ density profiles from an updated Saturn Thermosphere Ionosphere General Circulation Model^24^, as shown in Fig. 6. The model profiles are fitted hydrostatically to the ${H}_{2}$ densities observed by the INMS. To minimize the impact of changing latitude during the flyby, only measurements within 100 km of closest approach are used. The local deviations from the 1 bar surface (defined by gravity models^11,12^) are minimal and are expected to shift the whole altitude scale rather than affecting the bottom limit alone.

The electron temperature (${T}_{e}$) is assumed to remain constant below the closest approach (i.e., set to the closest approach value). Introducing an artificial decrease of ${T}_{e}$ similar to a modelled profile^51^ does not change the lower limit of the dynamo region more than the conservative uncertainties due to the measured densities and modelled temperatures of background atmosphere. The uncertainties in all the measured parameters are incorporated into the total error.

Because the ${H}_{2}$ profiles are extrapolated by means of a hydrostatic fit to the measured ${H}_{2}$ densities using only the bottom part of the profile (<100 km above closest approach) and due to the narrow band of latitudes covered by the profiles, the local deviations from the 1 bar surface (now defined by gravity models as mentioned above) are minimal and are also expected to shift the whole altitude scale rather than affecting the bottom limit alone.

### Negative Hall conductivity condition

A condition for the negative Hall conductivity can be derived by simplifying the expression for the Hall conductivity in Eq. (1). Using ${q}_{d}={Z}_{d}{q}_{e}$, quasineutrality ${Z}_{d}{n}_{d}={n}_{i}-{n}_{e}$, and ${\Omega }_{e}\gg {\nu }_{e}$ (in the dynamo region) we have:

7 $${{\rm{\sigma }}}_{H}\approx \frac{{{\rm{q}}}_{{\rm{e}}}}{B}\left[{n}_{e}+({n}_{i}-{n}_{e})\frac{{\Omega }_{d}^{2}}{{\nu }_{d}^{2}+{\Omega }_{d}^{2}}-{n}_{i}\frac{{\Omega }_{i}^{2}}{{\nu }_{i}^{2}+{\Omega }_{i}^{2}}\right].$$

The Hall conductivity becomes negative when

8 $${n}_{e}+({n}_{i}-{n}_{e})\frac{{\Omega }_{d}^{2}}{{\nu }_{d}^{2}+{\Omega }_{d}^{2}}-{n}_{i}\frac{{\Omega }_{i}^{2}}{{\nu }_{i}^{2}+{\Omega }_{i}^{2}} < 0,$$

or$$\frac{{n}_{e}}{{n}_{i}} < K$$, where

9 $$K({m}_{i},{m}_{d})=\left(\frac{{\Omega }_{i}^{2}}{{\nu }_{i}^{2}+{\Omega }_{i}^{2}}-\frac{{\Omega }_{d}^{2}}{{\nu }_{d}^{2}+{\Omega }_{d}^{2}}\right){\left(1-\frac{{\Omega }_{d}^{2}}{{\nu }_{d}^{2}+{\Omega }_{d}^{2}}\right)}^{-1}.$$

**Figure 11:**
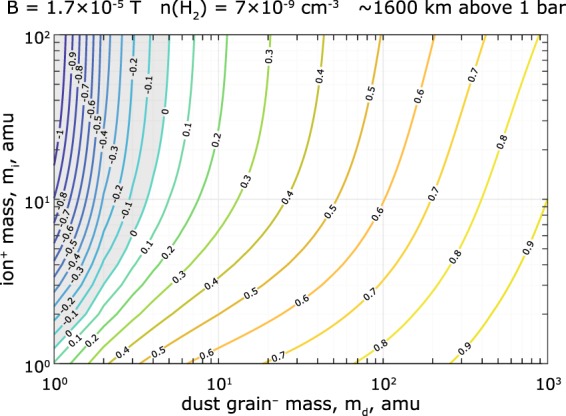
Contour plot of parameter $K({m}_{i},{m}_{d})$ such that ${\sigma }_{H} < 0$ for ${n}_{e}/{n}_{i} < K$, for a case of near-equatorial ionosphere of Saturn at ~1600 km above 1 bar. Shaded area marks the region where ${\sigma }_{H}$ is always positive.

Note that this is a general expression for any ionosphere-like plasma. For Saturn’s near-equatorial ionosphere at about 1600 km above 1 bar, a contour plot of $K({m}_{i},{m}_{d})$ is shown in Fig. 11, using typical values of $n({H}_{2})\approx 7\times {10}^{9}$ cm^−3^ and $|B|\approx 1.7\times {10}^{-5}$. Since ${m}_{d}\gg {m}_{i}$ and the the electrons are depleted by more than 70% (${n}_{e}/{n}_{i} < 0.3$) below 2000 km altitude^1^, the Hall conductivity is typically negative in the dynamo region.
